# Supplementary material for: Dynamic transcriptomic profiles of zebrafish gills in response to zinc supplementation
Source: BMC Genomics. 2010 Oct 11;11:553. doi: 10.1186/1471-2164-11-553 (PMC3091702; doi:10.1186/1471-2164-11-553)
Supplement: Additional file 2 — Interactive Direct Interaction Network representing the molecular interactions between zinc, copper, iron, calcium and proteins encoded by transcripts changed by zinc supplementation. Mini web-site containing index.html and hyperlinked pages in subdirectory describing a Direct Interaction Network automatically generated based on curated interactions contained within the proprietary PathwayArchitect database. Ovals represent proteins and the circles symbolize metal ions. Objects are coloured by their abundance in zebrafish at the time-point they were significantly different from the control is a scale from -4 fold (dark green) to +4 fold (dark red). Where significant differences were found at more than one time-point, the colour overlay shows expression at the first instance. Dark blue squares denote 'binding', and light blue squares 'expression'; green squares stand for 'regulation', green diamonds for 'metabolism', and green circles for 'promoter binding'. Arrow heads indicate directionality of the interaction where annotated. All nodes and edges can be further interrogated by selecting the relative area of the image. [file 1471-2164-11-553-S2.zip › PathwayArchitect Zn xs DIN/1594559.html]

# BINDING:

|  |  |
| --- | --- |
| Type | BINDING |
| Effect | None |


---

|  |  |
| --- | --- |
| Score | 0 |


---

|  |  |
| --- | --- |
| Reference Count | 2 |


---

|  |  |
| --- | --- |
| Mechanism | Unknown |


---

|  |  |
| --- | --- |
| Reference:0 || PMID | 2674939 |
| SourceID | 234652 |
| Species | Human |
| Experimental Condition | in-vitro |
| Description | The three dimensional structure of Mutant Human Lysozyme (A83kQ86DA92D) was determined by crystallography. The crystal was grown by hanging-drop and vapour diffusion using Lysozyme C, Phosphate, Sodium Chloride, Calci Chloride pH 6.0, at 293K. X-ray data were collected using Rotating Anode as a radiation source and were 77.0% complete to a resolution limit of 1.800 angstroms with an overall Rsym of 4.1%. This crystal contained 4 chains per asymmetric unit and belonged to the space group P 1 21 1. The structure was determined using Molecular Replacement and the modeled structure was refined to an R-factor = 17.2%. PDB: 1I22, MMDB: 15622. |
| Detection Method | three-dimensional-structure |
| Source | BIND |
  |
|  |  |


---

|  |  |
| --- | --- |
 Reference:1 || PMID | 2674939 |
| SourceID | 222266 |
| Species | Human |
| Experimental Condition | in-vitro |
| Description | The three dimensional structure of Lysozyme (E.C.3.2.1.17) (Holo) (Mutant with Gln 86 Replaced By Asp and Ala 92 Replaced By Asp) (Q86D,A92D) was determined by crystallography. X-ray data were collected using radiation to a resolution limit of 1.8 angstroms. The modeled structure was refined to an R-factor = 16.5%. PDB: 3LHM, MMDB: 3066. |
| Detection Method | three-dimensional-structure |
| Source | BIND |
  |


---

|  |  |
| --- | --- |
